# Comparison of the Usability of Apple M2 and M1 Processors for Various Machine Learning Tasks

**DOI:** 10.3390/s23125424

**Published:** 2023-06-08

**Authors:** David Kasperek, Pawel Antonowicz, Marek Baranowski, Marta Sokolowska, Michal Podpora

**Affiliations:** Department of Computer Science, Opole University of Technology, Proszkowska 76, 45-758 Opole, Polandmichal.podpora@gmail.com (M.P.)

**Keywords:** machine learning, deep learning, neural processing unit, neural processing cores, NPU benchmark, processor architectures, Apple M1, Apple M2, CoreML, neural engine

## Abstract

This paper compares the usability of various Apple MacBook Pro laptops were tested for basic machine learning research applications, including text-based, vision-based, and tabular data. Four tests/benchmarks were conducted using four different MacBook Pro models—M1, M1 Pro, M2, and M2 Pro. A script written in Swift was used to train and evaluate four machine learning models using the Create ML framework, and the process was repeated three times. The script also measured performance metrics, including time results. The results were presented in tables, allowing for a comparison of the performance of each device and the impact of their hardware architectures.

## 1. Introduction

In the current age, artificial intelligence algorithms are becoming more and more omnipresent, not only in robotic applications, but also in a wide range of application areas [1,2,3]. From ads and video recommendations [4,5,6], through text auto completion [7,8], to algorithms capable of producing award-winning art [9,10], an increasing number of people are using deep learning (DL) models in their work [11,12,13,14]. The production cycle of a deep learning model is time consuming and preferably requires understanding of complex concepts such as deep neural networks, neural network topology, training, and validation [15,16,17], as well as the application field, e.g., computer vision or natural language processing. Having that knowledge, training a production-capable model requires a lot of data [15,18,19] (or, alternatively, the use of transfer learning [18,19,20], which requires the knowledge of where to find such a model, and which one to use). Moreover, it is essential to have proficiency in using DL frameworks such as TensorFlow [21] or PyTorch [22]. Learning to create a good model [23] requires an investment of a considerable amount of time (preferably introduced at an early stage of education [24]) and knowledge of the basics of model preparation.

The authors have noticed that the process of learning and experimenting with machine learning for many researchers, students, or professionals is often preceded or accompanied by a difficult question—which hardware platform to choose [25,26,27,28]. This multi-factor optimization always includes an economical aspect [12,29], but the computational capabilities are not inessential [30,31]. Proper evaluation of the ‘money-to-value’ assessment is actively hindered by a ‘marketing fog’ [32,33], which tries to make the choice emotion-based instead of being based on any measurable factors.

Choosing a purposeful notebook for both everyday work and DL-oriented research is difficult. The general rule-of-thumb (better CPU, more RAM memory, modest graphics card) might still be a valid intuition-based choice; however, the evolution of CPUs brought a new player to the game: ARM-based (ARM—Advanced RISC Machine) ‘Apple M1’ chip (and its newer versions) [34,35,36,37], equipped with specialized GPU cores and NPU (Neural Processing Unit) cores. The presence of NPU cores sounds especially promising; however, not much evidence of the actual computational benefits is available. For this reason, the authors have decided to design and conduct a series of typical DL-related models/tasks, based upon readily available datasets, to evaluate and compare the new processors.

In this article, the authors verify the validity of Apple’s Deep Learning framework for some of the common DL challenges— image classification and regression using the Animals dataset (available on Kaggle’s webpage [38], consisting of over 29,000 images), image classification using a custom-made mini-dataset of 24 photos, a tabular dataset (the Kaggle’s Payment Fraud Detection Dataset [39]), and text-based use case—the Kaggle’s Steam Reviews [40] dataset.

### 1.1. Motivation

Deep learning researchers and enthusiasts worldwide are keen to obtain knowledge concerning new hardware that is affordable and could potentially speed up their experimentation with models. The marketing language is often not specific enough to explain the performance of the product. While, for most people, Neural Processing Unit performance is not the most important aspect of the laptop, for those who intend to work on deep learning, it could be a deciding matter. Moreover, the knowledge of the performance of particular hardware models at specific price points could be beneficial in terms of deciding whether to buy the more expensive chip or not. Having clear information about current hardware capabilities, especially the newest ones, may be of great interest for researchers, who have to decide on their next project, its budget, and its scope. Although it is feasible to carry out basic ML experiments on contemporary computers, the authors aim to delve into and juxtapose the “ML usability” of the aforementioned hardware platforms. In this context, “usability” is comprehended and examined as a quantitative assessment derived from employing systematic research methodologies for time-based evaluation of specific hardware platforms in preliminary machine learning experiments. The primary criterion under scrutiny is the computation time; nonetheless, it is advisable for the reader to expand the benefits of reading this paper by also taking into consideration the current prices of respective models.

This study is intended to deliver reliable information and arguments to scientists and enthusiasts who are interested in purchasing a new notebook equipped with hardware capable of accelerating neural network computations.

### 1.2. Scope and Limitations

The intended readership of this study comprises scientists and practitioners of deep learning who are considering purchasing an Apple laptop for their research, rather than investing in specialized High-Performance Computing (HPC) or Workstation equipment. Apple’s processors with a Neural Processing Unit (NPU) are marketed as ones that are indeed capable of accelerating model computations. Therefore, it is reasonable to compare only laptops that are equipped with Apple’s NPU.

Authors of this study compared Apple’s M-chip CPU family, including M1, M1 Pro, M2, and M2 Pro (details regarding exact hardware specifications are included in Section 2.4). Research was conducted using the same operating system (latest available to date, which was macOS Ventura 13.2) to introduce as little potential interference as possible. Additionally, Section 3.2 presents an alternative comparison–three different macOS versions whilst using the same hardware. All tests were designed to be used with the same code, environment, framework, and libraries (see Section 2.4) for all processors tested.

The comparison was performed using the Create ML framework [41] developed by Apple, designed and implemented with full compatibility and maximum efficiency of the CPU/hardware. Comparison of other DL frameworks may also be interesting, but, since it is strongly dependent on the availability of hardware support for the Apple M1/M2 chip as well as a proper implementation of the CPU extensions within a particular framework, a fair comparison is not yet possible.

While it would be interesting to measure the performance differences using statistical analysis, this work focuses primarily on the processing time of the datasets, as well as training and evaluation time of models created by Create ML.

### 1.3. Performance Measurements

The performance measurements were conducted with the usage of specialized software that stress-tested the hardware’s computational capabilities. The authors utilized common deep learning problems such as computer vision (classification) and regression, while using popular real-world datasets to test the viability of Apple’s chips in the tasks presented in Section 2.3. The proper analyses, as well as the correct interpretation of results, are key after collecting enough measurements on a particular hardware platform. The results wre converted and visualized in an easy-to-read and understandable form.

## 2. Materials and Methods

To ensure the repeatability and the ease of implementation of the models and datasets used within the research, the authors opted for the most native choices for the macOS-based platforms, which were readily available with fairly low entry threshold. The analyses and comparisons were implemented using the Swift programming language [42], Xcode Integrated Development Environment (IDE) [43], Xcode Playground (part of the Xcode IDE, introduced in 2014 [44], especially useful for rapid prototyping), and Create ML [41] (merged into a unified Apple ecosystem for creating, managing, and using machine learning models, with full support of the available hardware acceleration [45], as well as the ability to deploy the models onto mobile platforms).

Swift is a high-level programming language developed by Apple, released in 2014 as a replacement for Objective-C. It is commonly used as the first-choice language for applications built for Apple platforms [42].

Xcode is an integrated development environment (IDE) designed by Apple for developing software for macOS, iOS, iPadOS, watchOS, and tvOS. It includes a suite of tools for developing software, and also provides access to a wide range of Software Development Kits (SDKs) and Application Programming Interfaces (APIs) that are required for building applications for Apple’s platforms [43].

Xcode Playground is an interactive programming environment that allows developers to experiment with Swift code in an interactive way. It provides a lightweight environment for writing and running Swift code with live code execution. Xcode Playground is integrated within the Xcode IDE [44].

Create ML [41] is a framework and a collection of components and tools intended for easy preparation of machine learning models as well as their easy integration into custom applications. It features a GUI-based application for creating and training a model, which can be later distributed and used on other devices, including mobile applications. Create ML implements the Core ML framework to be able to benefit from the hardware it targets.

Core ML is Apple’s machine learning framework [45], designed specifically to benefit from the hardware acceleration capabilities of processors used in Apple devices. The Core ML framework is said to enable optimization of on-device performance by also using the GPU, NPU (named Neural Engine), and optimization of memory usage and power consumption [45].

### 2.1. Model Creation

The measurements were conducted using the ‘Benchmarker.playground’ script, available (open-source) in [46]. For a detailed insight into how the experiment was carried out, please refer to [37]. Within the ‘Benchmarker.playground’ script, each model was created by the use of custom functions written in the Swift programming language. The appropriate datasets were passed as arguments, and the trained models were returned. Finally, the models were tested using custom testing functions. The execution time of each stage and function was measured, which allowed the comparison of devices on which the script was running to be made. All the results were logged into the console output. The process of training and evaluation of all models was repeated three times.

### 2.2. Model Export as .mlmodel

Models created with the use of Create ML, (whether implemented in the Playground sandbox or in an actual application), can be easily exported as a file [47]. This is carried out using Apple’s Core ML framework file format—an ‘*.mlmodel’ file [48].

The .mlmodel file contains the prediction methods of a machine learning model, including all of its configuration data and metadata [49]. These parameters were previously extracted from its training environment and then processed to be optimized for Apple device performance [50].

The ‘.mlmodel’ file includes the following sections [51]:

1. Metadata—Defines the model’s metadata;
2. Interface—Defines the input and output features;
3. Architecture—Encodes the model’s architecture;
4. Parameters—Stores all values extracted during the model training.

To correctly interpret the input data and produce valid output predictions, features need to be defined and specified in the .mlmodel [51,52]. This includes “Metadata”, such as the author, license, and model version, stored in the form of a dictionary [51,53,54]. The “Model description” information such as the names, data types, and shapes of the features are saved in the “Interface” module [51,52,54].

In the next step, the architecture of the model needs to be defined [51]. This involves the definition of the model’s structure, including the number and type of layers, the activation functions, and other operations [50,51].

Then, the model parameters are defined [55]. These are values of variables and coefficients, including weights and biases of each layer [51,55,56].

The model metadata, interface, architecture, and parameters are encoded into a data structure called ‘protobuf message definitions’ [51,54,55]. The Protocol Buffer syntax allows encoding and decoding information about the Core ML model in any language that supports the Protocol Buffer serialization technology, (including Python, C++, C#, or Java) [54,55]. ‘Model.proto’ is the essential file of the Core ML Model *protobuff message definitions* [57]. It describes the structure of the model, the type of inputs and outputs it can have, and metadata [54,57]. The file also includes the ‘specification version’, which determines the versions of Core ML format specification and functionalities that it can provide [54,55,57,58]. Each version of the target device’s operating system has its own way of implementing the model, so it is crucial to also include these in the model specification [58].

All of the Model’s *protobuff message definitions* are encoded into binary format, which can be deployed on Apple platforms and encoded while loading the model [51,55].

### 2.3. Datasets Used

The authors used four distinct datasets for their study. These datasets were used for different purposes: two for image classification, one for tabular classification, and one for tabular regression. Of these, one dataset was created by the authors, and the rest were obtained from the Kaggle [59] website.

#### 2.3.1. The Animals Dataset

The Animals dataset [38] was downloaded from Kaggle.com [59]. It contains 29,071 images divided into a training subset and testing subset. It is published under a Creative Commons license. Images are in different shapes, have three colour channels, and animals often are only partially visible in the picture (e.g., only the head of an ostrich), while in other cases, the whole body is portrayed. To set the image size for the network properly, it is essential to know what objects are in the images. One picture could be presenting a large animal (e.g., an elephant), but from a large distance so that it appearz small, while another could be a picture of a shrimp, but taken from a close distance and zoomed in. Knowing that the depicted objects may vary in size, and the classes can be similar enough that differentiating between them requires a certain level of detail, it becomes justified to increase the input size of the neural network. Hence, it is important to take a good look at the data.

**Figure 1:**
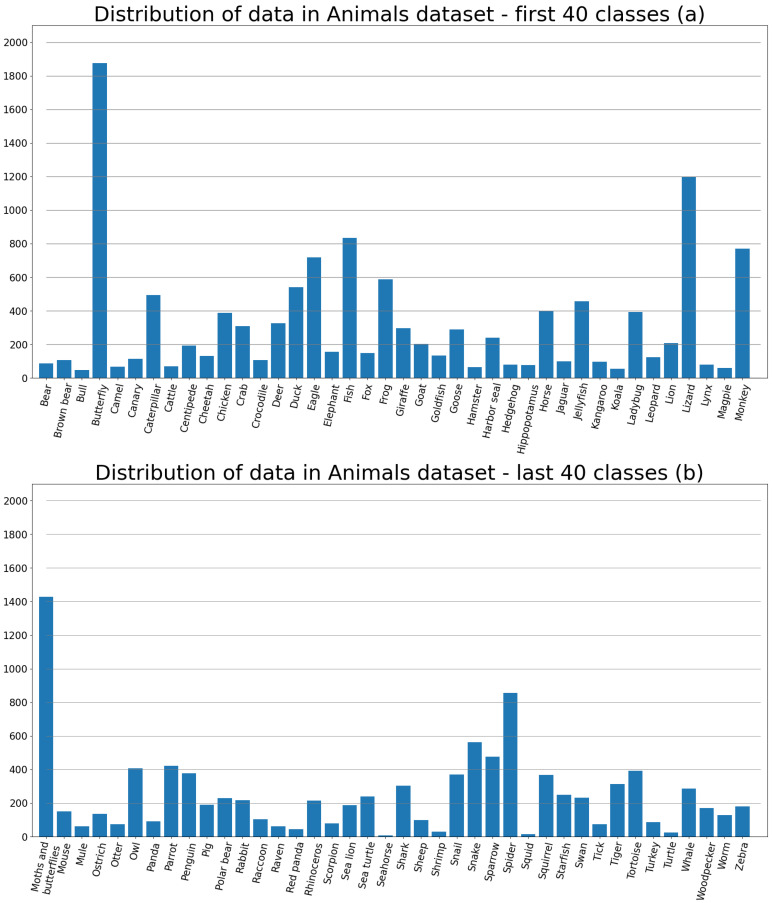
(**a**)—The distribution of data of the first 40 classes, (**b**)—the distribution of the remaining 40 classes.

**Figure 2:**
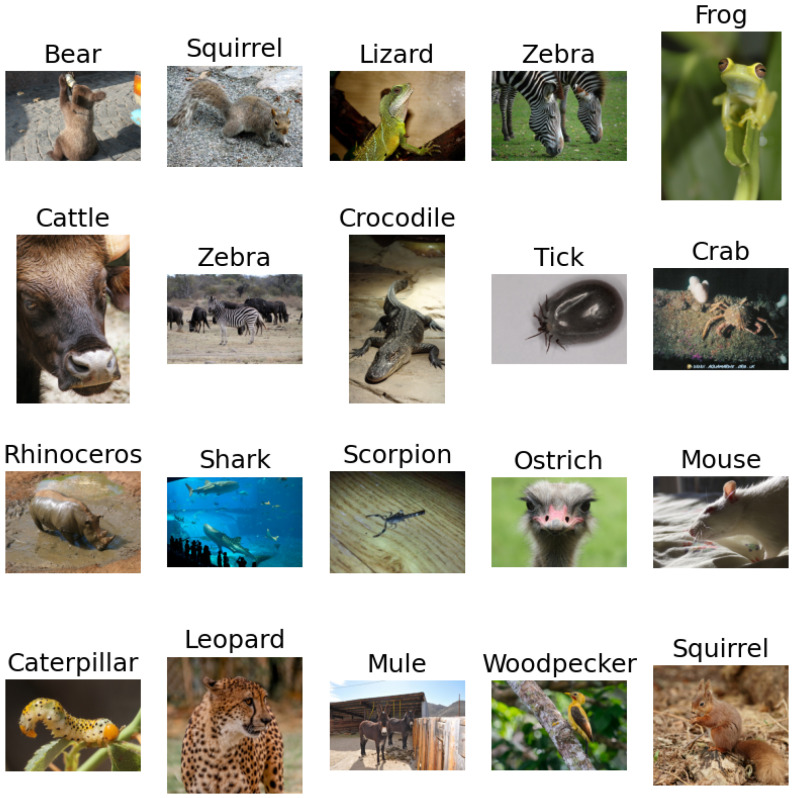
A sample of 20 random images from the Animals dataset [38] subset.

The training set consists of 22,566 images divided into 80 classes, making the problem a multiclass classification. The distribution of data in the training subset is presented in Figure 1. The testing set is made of 6505 images. Figure 2 portrays a random sample of 20 pictures of different classes from the Animals dataset.

#### 2.3.2. The Payment Fraud Detection Dataset

The Online Payments Fraud Detection Dataset was published on Kaggle under Creative Commons Attribution-NonCommercial-ShareAlike 4.0 International license. The dataset consists of 5,080,807 entries in a “.csv” file, which translates into 493.5 MB. The data are divided into two classes (fraud or non-fraud), making it a binary classification problem. Every entry has nine features, as explained on the Kaggle’s dataset webpage [39].

#### 2.3.3. The Steam Reviews Dataset

The Steam Reviews Dataset 2021, obtained from Kaggle, represents the most recent dataset used in this study. It comprises approximately 21 million user reviews pertaining to approximately 300 games on the Steam platform. The dataset is available under the GNU GPL 2 license. To prepare the dataset for utilization in “Create ML”, the authors conducted a data cleaning process using the Python language, along with the “Pandas” library. The cleaning procedure involved removing specific columns such as “comment text”, “author”, “creation date”, and others. The resulting cleaned dataset was saved as “SteamReviewsCleaned.csv”, resulting in a reduction in size from 3 GB to 2.15 GB. This dataset was utilized for a tabular regression problem within the context of this study.

#### 2.3.4. The ClassifierData Dataset

**Figure 3:**
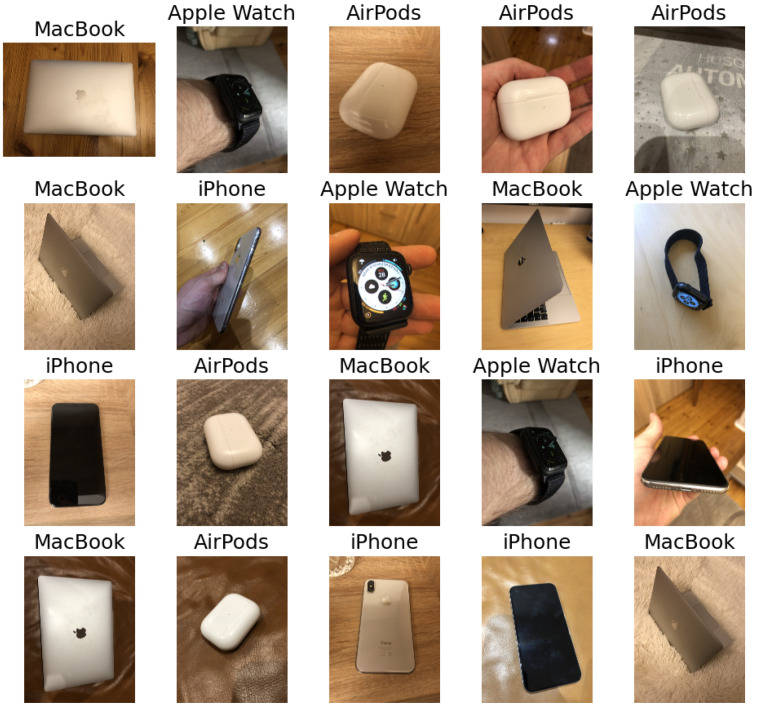
A random sample of 20 images from the ClassifierData training subset.

**Figure 4:**
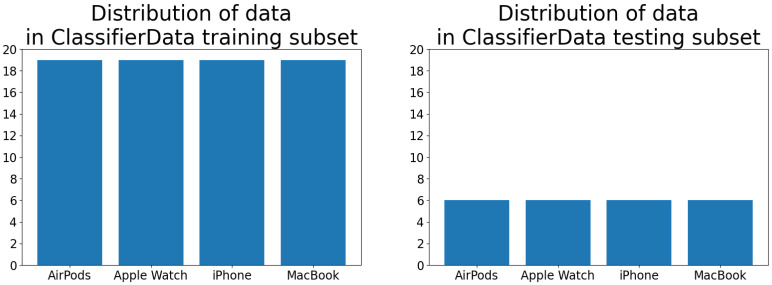
Distribution of data in training (**left**) and testing (**right**) subsets of the ClassifierData dataset.

This very small custom dataset was created by one of the authors. It was composed of four classes: “IPhone”, “MacBook”, “Apple Watch”, and “AirPods”. Every class contained 25 photos—19 in the training subset and 6 in the test subset. Every image was taken from different angles as well as in varyied lighting. Some pictures were taken of objects held in hand, while others were taken while lying on the floor, table, or carpet. Similarly to the Animals dataset, Figure 3 presents a random sample of images from all four classes. The data distribution of both training and test subsets is visualized in Figure 4. The subset was structured in a Create ML-compliant format [47], i.e., as image files placed inside the class-related folders. All images were sampled using a mobile device, photographing the target in various angles and light conditions. 

### 2.4. Framework and Hardware Used in the Trials

The study was conducted on four notebooks, each one running the macOS Ventura operating system version 13.2 [60], with the Xcode Integrated Development Environment, version 14.2 [61].

The primary objective of the research was to investigate the usability of modern laptops equipped with ARM-based M1/M2-series CPUs in popular machine learning tasks. Since the manufacturer of M1- and M2-equipped laptops declares that the presence of the NPU cores in the CPUs makes them useful and interesting in machine learning applications, and since there are many researchers willing to buy a suitable portable platform for everyday work, the authors have decided that it may be worthwhile and interesting to put the eligibility of the NPU-equipped CPUs in DL tasks to a test.

All examined machines were ARM-based Apple MacBook Pro notebooks.

The first tested computer was the 2020 M1 MacBook Pro. The model started Apple’s transition from Intel to ARM architecture [34]. It was equipped with the first version of the Apple M1 chip. The CPU included four high-performance and four energy-efficient cores. The chip was also equipped with an eight-core GPU. The first version of Neural Engine—a 16-core neural processing unit (NPU) —was also included in this chip [34]. The device had 8 GB of RAM. It is referred to as ‘M1’ in this work.

Another device from the M1-series was the MacBook Pro 2021. It included the strengthened version of the M1 Pro chip, which also included an eight-core CPU, but the allocation of the cores differed: six were high-performance and two were energy-saving.

The M2 series processor is an upgraded version of the previous M1 processor, boasting a speed increase of about 40%. It features eight cores, which are designed to be four performance cores and four efficiency cores [35]. Additionally, the processor includes 10 GPU cores and 16 Neural Engine cores. The M2-equipped laptop used in the research had 16 GB of RAM.

The fourth laptop used for the research was a MacBook Pro equipped with an Apple M2 Pro processor and 16 GB of RAM. This processor was composed of 10 cores, with 6 of them being performance cores and 4 being efficiency cores [36]. The processor also included 16 GPU cores and 16 Neural Engine cores.

## 3. Results

The most important result presented within this paper is the comparison of the computational performance of the M1 and M2 processors in ML tasks, presented in Section 3.1 and discussed in Section 4 and Section 5. The comparison is made based on the performance (processing time) of ML models included within the ‘Benchmarker.playground’ project.

Section 3.2 includes an additional, also interesting, analysis of a possible impact of the versions of macOS and Xcode on the ML tasks’ processing time.

### 3.1. Measurement of the Impact of the Processor Model on the Model Creation Time

During the research, three measurements of model training and testing time were performed. The computer was consistently connected to a power source throughout the script execution to ensure uninterrupted performance and avoid any potential limitations caused by energy-saving features. This allowed us to benchmark the efficiency of M-series processors in machine learning tasks using Apple’s ecosystem.

#### 3.1.1. Running the Benchmark

The measurements were performed on four computers. The ‘Benchmarker.playground’ file was copied to the hard drive of each machine. Then, the file was opened in the Xcode environment and executed. During the tests, each computer was left without any additional tasks. When the running of the script had finished, the console output was saved to a ‘.txt’ file.

**Figure 5:**
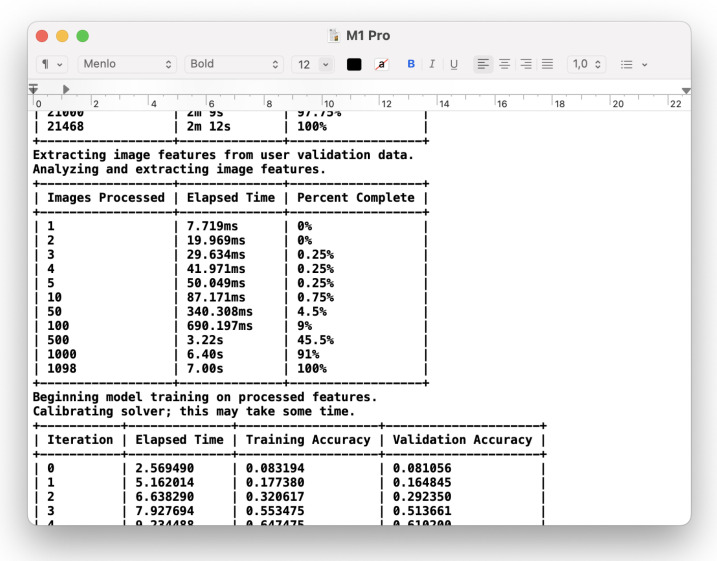
A part of the benchmark report of the ‘M1 Pro’ computer.

A screenshot of the ‘M1 Pro.txt’ file with the console output log from the ‘Benchmarker’ playground executed on the M1 Pro Mac is presented in Figure 5.

#### 3.1.2. The Results of the Benchmark

The results of the benchmark performed on the ClassifierData dataset were similar in terms of overall time, except for the M1 Pro, which was over two times slower than the other processors. Each training took, on average, a different number of iterations (epochs); the means spanned from 11 to 14.667. The M1 achieved the best average result by a slight margin, outperforming the second-fastest (which was the M2 Pro) by 383 ms. The third average result was achieved by M2, which lost about 70 ms to the Pro variant. The worst performance on the ClassifierData dataset was the M1 Pro; despite taking the second-lowest average number of iterations (11.333), it scored by far the worst time of 8.622. Every model trained on each chip achieved 100% for both training and validation accuracy. This test was the quickest one due to the small size of the dataset.

Table 1 shows the time and accuracy results of the model training and testing process, performed on the ‘ClassifierData’ dataset using Create ML.

The multiclass classification test was performed by utilizing the Animals dataset of over 29,000 images, split into training and testing subsets. The results of the benchmark are presented in Table 2. The quickest of all tested processors while training the model on the Animals dataset was M2 Pro. It took the M2 Pro 169.7 s to complete the test. The second-fastest processor, (M2), took 186.689 s to train the model, which is 9% slower than the Pro variant; however, the evaluation time was basically the same, at 40.796 for the Pro and 40.796 for the basic M2. The M1 Pro finished the training process in 193.219 s, which earned it third place. This result is 12% slower than the M2 Pro. The evaluation time was approximately 5 s slower than both M2 and M2 Pro. The slowest one, M1, achieved a result of 236.285 s. It was 28% slower than the fastest processor; simultaneously, it was the only one that completed the training in over 200 s on average. The evaluation also took the longest, exceeding 47 s. All processors achieved similar training and validation accuracy, of about 88% and 86.5%, respectively.

Upon examining the results of the benchmark conducted on the PaymentFraud dataset displayed in Table 3, it is apparent that the accuracy levels for all tested cases were comparable, with minor disparities emerging during the data analysis phase. During this stage, the M2 Pro processor exhibited the quickest performance, taking only 1.924 s, while the slowest was the M1 at 2.310 s. The M2 processor, on the other hand, completed the data analysis in 2.01 s, and the M1 Pro required 2.161 s.

The most notable differences in processing time were found during the overall model building phase. The M2 processor was the speediest in this regard, finishing the model building task in 102.109 s. The M1 processor took 13% longer, completing the same assignment in 117.546 s, while the Pro version of the M1 took 146.641 s, which was 43% slower than the M2 processor. Interestingly, in this case, the M2 Pro processor proved to be the slowest, taking 151.659 s to complete the task, which was 48% slower than the M2’s base version.

Table 4 displays the benchmark outcomes for the SteamReviewCleaned dataset. Noticeably, the table does not present the maximum error and root-mean-square error findings for the training, validation, and test data. These results are excluded due to their consistency across all cases, as was shown in our preceding publication [37].

The M2 Pro processor boasted the swiftest processing time, taking only 7.981 s to complete the task, while the M1 Pro and M2 processors processed the data in nearly the same amount of time, clocking in at 8.143 s and 8.255 s, respectively. Meanwhile, the M1 proved to be the slowest, taking 9.276 s.

During the model building phase, the M2 Pro processor was once again the fastest, completing the task in only 12.545 s. The M2 processor followed closely, requiring 13.395 s to build the model. The M1 Pro took 14.713 s to finish the task, while the M1 took 15.545 s. With the exception of the M1, which took 2.023 s, all processors required less than 1.75 s to evaluate the model. The M2 Pro processor was once again the quickest in this task, taking only 1.596 s, while the M1 Pro and M2 processors achieved similar times of 1.736 and 1.665 s, respectively.

Table 5 displays the execution times of a script on various tested platforms. The MacBook with an M2 processor completed the entire script in the fastest time, taking 1088.989 s, with an average of 362.996 s per iteration. All iterations took a similar amount of time, with the fastest iteration completed in 359.398 s and the slowest in 364.796 s. The MacBook with an M2 Pro processor took 9% longer to execute the script, taking 1186.557 s, with an average of 395.519 s per iteration, and the slowest iteration took 397.401 s. The MacBook with an M1 Pro processor took 1281.761 s, with an average of 427.254 s per iteration, which was 18% longer than the M2 MacBook. The MacBook with M1 took the longest time, taking 1314.943 s, with an average of 438.314 s per iteration. This took 21% longer than the fastest tested MacBook. The fastest iteration took 436.706 s, and the slowest iteration took 439.858 s on the slowest MacBook tested.

### 3.2. Measurement of the Impact of the macOS Version on the Model Creation Time

An evaluation of the influence of the macOS wersion on the script execution time was carried out on the MacBook Pro referenced as ‘M1 Pro’. The measurement was performed on three various versions of macOS and Xcode IDE:

- macOS Monterey 12.4 and Xcode 13.4;
- macOS Ventura 13.0.1 and Xcode 14.2;
- macOS Ventura 13.2 and Xcode 14.2.

The computer was not used during each execution of the script. The device remained connected to the power source at all times to prevent any limitations due to energy-saving features.

Table 6 displays the benchmark results for various versions of systems and Xcode for the ClassifierData dataset. The Macbook with macOS 12.4 and Xcode 13.4 installed achieved the fastest training dataset, finishing the task in 6.957 s. A very similar time was recorded on a Macbook with macOS 13.2 and installed Xcode 14.2, at 7.083 s. The slowest was macOS 13.0.1, taking 7.573 s to complete the task. However, when analyzing the validation set, macOS 13.2 Macbook was the fastest, taking merely 0.912 s. The other platforms analyzed the validation set at a similar time, with macOS 12.4 and 13.0.1 completing the task in 0.921 and 0.922 s, respectively. The macOS 13.2 performed the fastest training, with the entire training taking 0.214 s, attaining completion after 11.3 iterations. The training took 0.217 s on a Macbook with macOS 12.4 after 12 iterations. The longest training, lasting 0.263 s, was on macOS 13.0.1, ending after 14 iterations. In all cases, the model achieved 100% accuracy on the training and validation sets. Overall, the entire process of analyzing the sets and building the model was the fastest on a Macbook with macOS 12.4, taking 8.571 s. A few milliseconds longer, the task was completed on a Macbook with macOS 13.2 in 8.662 s. The Macbook with macOS 13.0.1 took the longest at 9.175 s. The test set was analyzed the fastest on a Macbook with macOS 12.4, completing the task in 1.993 s. On a Macbook with macOS 13.0.1, the task was completed in 2.093 s. This task took the longest on a Macbook with macOS 13.2, requiring 2.203 s. The evaluation of the entire model on the test set was the fastest on a Macbook with macOS 12.4, taking 2.138 s, and the previously prepared model on this version achieved 95.83% accuracy. The same accuracy was achieved by the model prepared for macOS 13.0.1, but it took 2.309 s. The best accuracy was achieved on macOS 13.2, at 98.61%, but evaluating the model on the test set required 2.421 s.

Table 7 presents the development times of the model for the Animals set using different versions of macOS and Xcode. The analysis of the training set took the longest time on macOS 12.4, i.e., 137.667 s. The Macbook with macOS 13.2 analyzed the set for 132.333 s, while macOS 13.0.1 achieved the best result by completing the task in 130.667 s. The validation set was analyzed for 7.16 s on macOS 12.4 and 7.043 s on macOS 13.2, while the shortest time of 6.937 s was achieved on macOS 13.0.1. The model was trained for 40.424 s on a MacBook with macOS 12.4, achieving an accuracy of 87.91% for the training set and 86.12% for the validation set. The entire modeling process took 198.985 s. On macOS 13.0.1, the model was trained for 39.709 s, achieving an accuracy of 87.97% for the training set and 86.95% for the validation set. The entire modelling process took 191.641 s. For macOS 13.2, it took 193.219 s to build the entire model, with 39.442 s dedicated to training. In this case, the model achieved an accuracy of 88.24% for the training set and 86.64% for the validation set. The shortest time to evaluate the model on the test set was on a MacBook with macOS 13.2, taking 45.415 s, with an achieved accuracy of 84.84%. The model was evaluated on the test set in 46.095 s on macOS 13.0.1, with an accuracy of 84.87%. On a MacBook with macOS 12.4, the model was evaluated for the longest time of 46.590 s, achieving an accuracy of 84.94% for the test set.

Table 8 presents the results acquired for the PaymentFraud dataset. Each system analyzed the training set for a similar amount of time. The macOS 13.0.1 with Xcode 14.2 installed analyzed the fastest set in 2.153 s, and the slowest analysis was on macOS 13.2 with the same Xcode version in 2.161 s. The analysis on macOS 12.4 took 2.158 s. The accuracy of the test set was 99.96% on each tested system, with only minor differences. The highest accuracy of 99.99% was achieved on macOS 13.0.1, and the lowest accuracy of 99.95% was achieved on macOS 13.2. The model trained on macOS 12.4 achieved an accuracy of 99.97% for the same validation set. The fastest model was built on macOS 12.4 in 143.456 s, while the slowest model building process was on macOS 13.2 in 146.641 s. It took 144.639 s to build the entire model on macOS 13.0.1. The accuracy measurement for the test set took 1.298 s on macOS 12.4 and 1.292 s on macOS 13.0.1. The fastest accuracy measurement of the model was on macOS 13.2 in 1.279 s.

The training times for the SteamReviewsCleaned dataset on various versions of Macbook Pro with M1 Pro processor are displayed in Table 9. The quickest model was trained on macOS 13.2 equipped with Xcode 14.2, which took 14.713 s, with 8.143 s spent on analyzing the dataset. The longest training time was documented on macOS 13.0.1, where the whole process took 15.006 s, and dataset analysis took 8.356 s. The model evaluation process on different systems had similar timings, with macOS 13.0.1 having the longest evaluation time of 1.834 s and macOS 12.4 having the shortest evaluation time of 1.732 s. In the case of macOS 13.2, the evaluation process took 1.736 s.

Table 10 provides a summary of the benchmark results for different versions of macOS. The quickest benchmark was completed in 1276.076 s on a MacBook running macOS 13.0.1 with Xcode 14.2 installed. On average, each iteration took 425.359 s. The fastest iteration was completed in 421.602 s, while the slowest iteration took 432.384 s. The benchmark took the longest time to complete on a MacBook running macOS 12.4 with Xcode 13.4 installed, taking 1293.449 s to finish, with one iteration taking 431.150 s. In this case, the slowest iteration took 436.282 s, while the quickest iteration was completed in 431.150 s. On macOS 13.2, the benchmark took 1281.761 s to complete, with an average of 427.254 s needed for each iteration.

## 4. Discussion

The gathered results present the comparative computational performances of the Apple laptops equipped with four different M-family processors, including the most recent M2 Pro chip. All of the tested hardware (including the previous M1 generation) is perfectly capable of performing ML tasks that do not require processing millions of images or hundreds of gigabytes (or more) of data. Each and every dataset has been successfully analyzed and processed. Every created model had similar efficacy; regardless of the chip it was trained on, the results were satisfactory.

Three of the used datasets are available to the public; as well, the hardware and software specifications provided in this research ensure that the reproducibility and comparability of the results is possible for other researchers.

The chips were tested for machine learning applications with the use of Apple’s Create ML. A rather surprising average result is the overall performance of the M2 Pro variant. It was outperformed by the base variant by approximately 9%; however, this was mainly due to poor performance of the more expensive variant on the PaymentFraud dataset. This result may affect someone’s decision as to whether it is beneficial to increase their budget to buy the Pro chip or save money and buy the cheaper standard M2. The M1 Pro also had the same difficulties with the same dataset that its newer counterpart had, achieving a much worse time than the base version of the chip.

The research also included an evaluation of the script execution time on various macOS versions. The tasks were performed on the same MacBook Pro laptop, with different versions of the operating system and development environment.

The obtained results showed, that the version of the macOS has an impact on the script execution times. For the ‘ClassifierData’, almost all times were longer after updating the operating system from the previous ‘major version’ (macOS 12.4) to the next new ‘major version’ (macOS 13.0.1). However, the installation of a system update with bug fixes and improvements (macOS 13.2) decreased the execution time to a value which was comparable with the results from the previous ‘major version’.

In case of the ‘Animals’ dataset, the data analysis time also changed with the version of the operating system, with macOS 13.0.1 being the fastest. The model training time was comparable in each test, which suggests that system updates have no impact on the training process.

The tests performed on tabular datasets (‘PaymentFraud’ and ‘SteamReviewsCleaned’) showed no remarkable difference between the execution time of the training and evaluation process. This shows that differences are visible only when working with image datasets.

## 5. Conclusions

Upon conducting an in-depth analysis of the collected results, our study revealed significant findings that may provide insights into the performance of distinct chips when employed for training and testing models using Create ML.

The results presented in the paper demonstrate that the M2 chip may exhibit superior performance compared to the M2 Pro (as shown in Table 5), implying that the M2 chip may be a favorable choice for tasks demanding efficient model creation.

While it is difficult to provide a definitive recommendation for future processors or operating systems due to their evolving nature, in this study, the authors proposed a methodology for evaluating the effectiveness of specific hardware architectures, which can be investigated by the researchers themselves, using the proposed [46] benchmark.

The observations also reveal that the ‘Pro’ series of respective chips (namely, M1 Pro and M2 Pro) do not meet the anticipated time-related performance efficiency for model creation using the ‘Payment Fraud’ dataset (see Table 3). Moreover, the processing performance of the ‘M1 Pro’ chip proved to be below average for the small ‘ClassifierData’ dataset, whereas the ‘M1’ chip exhibited surprisingly good performance on the same dataset (see Table 1). These observations indicate that certain characteristics of datasets can indeed impact the performance of specific chip models. The research provided evidence that the expected superiority of the ‘Pro’ variant should be challenged for model training, even when using Apple’s own ‘Create ML.’

Lastly, the experimental comparative research resulted in the formulation of additional insights of minor significance: it was confirmed that the multiclass classification performance results were consistent with the CPU-generation-related expectations, and that the operating system version had an impact on the processing time, particularly in the case of image datasets.

The results presented within this study bring theoretical and managerial implications that extend beyond the immediate scope of hardware platform performance evaluation. The insights gained from comparing respective processors and their performance in machine learning tasks using Create ML shed light on the complexities and nuances of hardware-platform-specific characteristics. From a theoretical standpoint, these findings help to understand the impact of particular hardware choices on the efficiency and effectiveness of ML computation time. By examining the performance (and its variations across various chips), researchers can refine their theoretical models and develop more nuanced frameworks for leveraging the benefits of hardware acceleration for typical Machine Learning applications. On a managerial level, the research findings have substantial value for decision-makers who are considering hardware platforms for machine learning researchers and initiatives. The performance disparities observed among the tested chips help to highlight the benefits and importance of careful evaluation of the hardware requirements, based on the specific needs and characteristics of current machine learning projects.

### Future Work

The research conducted in this study opens avenues for further exploration in the realm of multi-platform analysis (the comparison of Apple platforms against other, non-Apple, platforms). While this research focused on the performance evaluation of hardware platforms utilizing primarily Create ML, future studies could extend the analysis to encompass a broader range of machine learning frameworks, especially the most popular ones—TensorFlow and PyTorch. By conducting experiments using TensorFlow and PyTorch across different hardware platforms and operating systems, a more comprehensive understanding of the performance variations and platform compatibility can be obtained. Additionally, investigating the impact of different frameworks on the efficiency and effectiveness of model creation would contribute valuable insights to the field. Such multi-platform approaches will provide a more comprehensive assessment of the hardware-platform-specific characteristics and guide researchers and practitioners in making informed decisions regarding the choice of frameworks and hardware configurations for their machine learning tasks.

## Figures and Tables

**Table 1:** Average results for the processor-related test performed using the ‘ClassifierData’ dataset.

	M1	M1 Pro	M2	M2 Pro
Training data—analysis time (s)	2.543	7.083	3.157	2.987
Validation data—analysis time (s)	0.346	0.912	0.366	0.402
Model training—total time (s)	0.213	0.214	0.261	0.256
Model training—number of iterations	11	11.333	14.667	12.333
Training Accuracy	100%	100%	100%	100%
Validation Accuracy	100%	100%	100%	100%
Total model creation time (s)	3.689	8.622	4.145	4.072
Evaluation data—analysis time (s)	0.824	2.203	0.876	0.949
Evaluation Accuracy	97.22%	98.6%	98.6%	95.8%
Total model evaluation time (s)	1.047	2.421	1.022	1.147

**Table 2:** Average results for the processor-related test performed using the ‘Animals’ dataset.

	M1	M1 Pro	M2	M2 Pro
Training data—analysis time (s)	132.333	132.333	116.667	116.333
Validation data—analysis time (s)	7.240	7.043	6.087	6.133
Model training—total time (s)	79.364	39.442	49.746	33.356
Training Accuracy	88.26%	88.24%	88.11%	88.13%
Validation Accuracy	85.98%	86.64%	86.13%	86.52%
Total model creation time (s)	236.285	193.219	186.689	169.777
Evaluation data—analysis time (s)	41.050	40.393	36.033	36.050
Evaluation Accuracy	84.87%	84.84%	84.91%	84.87%
Total model evaluation time (s)	47.284	45.415	40.768	40.796

**Table 3:** Average results for the processor-related test performed using the ‘PaymentFraud’ dataset.

	M1	M1 Pro	M2	M2 Pro
Data processing time (s)	2.310	2.161	2.010	1.924
Training accuracy	99.96%	99.96%	99.96%	99.96%
Validation accuracy	99.96%	99.95%	99.97%	99.96%
Total model creation time (s)	117.546	146.641	102.109	151.659
Evaluation accuracy	99.96%	99.96%	99.96%	99.95%
Total model evaluation time (s)	1.280	1.279	1.139	1.107

**Table 4:** Average results for the processor-related test performed using the ‘SteamReviewsCleaned’ dataset.

	M1	M1 Pro	M2	M2 Pro
Data processing time (s)	9.276	8.143	8.255	7.891
Total model creation time (s)	15.545	14.713	13.395	12.545
Total model evaluation time (s)	2.023	1.736	1.665	1.596

**Table 5:** Average iteration times of the ‘Benchmarker_Playground.playground’ program.

	M1	M1 Pro	M2	M2 Pro
Average iteration time (s)	438.314	427.254	362.996	395.519
Fastest iteration (s)	436.706	423.018	359.398	394.179
Slowest iteration (s)	439.858	431.328	364.796	397.401
Total measurement time (s)	1314.943	1281.761	1088.989	1186.557

**Table 6:** Average results for the OS-related test performed using the ‘ClassifierData’ dataset.

	macOS 12.4 Xcode 13.4	macOS 13.0.1 Xcode 14.2	macOS 13.2 Xcode 14.2
Training data—analysis time (s)	6.957	7.573	7.083
Validation data—analysis time (s)	0.921	0.922	0.912
Model training—total time (s)	0.217	0.263	0.214
Model training—number of iterations	12	14.7	11.3
Training Accuracy	100%	100%	100%
Validation Accuracy	100%	100%	100%
Total model creation time (s)	8.571	9.175	8.622
Evaluation data—analysis time (s)	1.933	2.093	2.203
Evaluation Accuracy	95.83%	95.83%	98.61%
Total model evaluation time (s)	2.138	2.309	2.421

**Table 7:** Average results for the OS-related test performed using the ‘Animals’ dataset.

	macOS 12.4 Xcode 13.4	macOS 13.0.1 Xcode 14.2	macOS 13.2 Xcode 14.2
Training data—analysis time (s)	137.667	130.667	132.333
Validation data—analysis time (s)	7.160	6.937	7.043
Model training—total time (s)	40.424	39.709	39.442
Training Accuracy	87.91%	87.97%	88.24%
Validation Accuracy	86.12%	86.95%	86.64%
Total model creation time (s)	198.985	191.641	193.219
Evaluation data—analysis time (s)	41.977	40.887	40.393
Evaluation Accuracy	84.94%	84.87%	84.84%
Total model evaluation time (s)	46.590	46.095	45.415

**Table 8:** Average results for the OS-related test performed using the ‘PaymentFraud’ dataset.

	macOS 12.4 Xcode 13.4	macOS 13.0.1 Xcode 14.2	macOS 13.2 Xcode 14.2
Data processing time (s)	2.158	2.153	2.161
Training accuracy	99.96%	99.96%	99.96%
Validation accuracy	99.97%	99.99%	99.95%
Total model creation time (s)	143.456	144.639	146.641
Evaluation accuracy	99.96%	99.96%	99.96%
Total model evaluation time (s)	1.298	1.292	1.279

**Table 9:** Average results for the OS-related test performed using the ‘SteamReviewsCleaned’ data-set.

	macOS 12.4 Xcode 13.4	macOS 13.0.1 Xcode 14.2	macOS 13.2 Xcode 14.2
Data processing time (s)	8.347	8.356	8.143
Total model creation time (s)	14.924	15.006	14.713
Total model evaluation time (s)	1.732	1.834	1.736

**Table 10:** Cumulative results of all OS-related tests performed.

	macOS 12.4 Xcode 13.4	macOS 13.0.1 Xcode 14.2	macOS 13.2 Xcode 14.2
Average iteration time (s)	431.150	425.359	427.254
Fastest iteration (s)	428.403	421.602	423.018
Slowest iteration (s)	436.282	432.384	431.328
Total measurement time (s)	1293.449	1276.076	1281.761

## Data Availability

The ‘Benchmarker.playground’ project, including all source code, is made available as Open Source on GitHub, at https://github.com/dKasperek/Benchmarker (accessed 3 March 2023). The code is implemented to run three iterations, each one creating four ML models, using the following datasets: ‘Animals’ (available from [38]), ‘PaymentFraud’ ([39]), ‘SteamReviews’ ([40]), and the ‘ClassifierData’ (available within the above-mentioned GitHub project). The datasets should be imported into the ‘Resources’ folder inside the Xcode Playground project.
